# Reinforcement Learning-Based Control of a 4-Wheel Independent Steering Mobile Robot for Robust Path Tracking in Outdoor Environments

**DOI:** 10.3390/s26061761

**Published:** 2026-03-10

**Authors:** Hyoseok Lee, Hyun-Min Joe

**Affiliations:** Department of Robot and Smart System Engineering, Kyungpook National University, Daegu 41566, Republic of Korea; erlangen416@gmail.com

**Keywords:** 4-wheel independent steering, mobile robot, path tracking, reinforcement learning, Sim-to-Real

## Abstract

This paper proposes a reinforcement learning (RL)-based control method for robust path tracking of a 4-wheel independent steering (4WIS) mobile robot in outdoor rough terrain environments. Traditional wheeled robots typically suffer from limitations including mobility constraints in narrow spaces, path deviations caused by ground slip, and reduced traction on rough terrain. To address these challenges, we designed a 4WIS mobile robot and implemented an architecture that independently controls the steering and driving of each wheel. The RL state space is defined by look-ahead path information, robot pose, velocity, and tracking errors, while the action space consists of target angular velocity and steering angle. To ensure robust performance, we applied random path and terrain generation and implemented domain randomization for sensors and actuators based on empirical GPS and motor data. The proposed controller was validated against the Pure Pursuit algorithm through dynamic simulations and real-world experiments. In simulations mimicking outdoor terrain, the controller reduced lateral and heading RMSE by 6.32% and 16.00%, respectively. In actual outdoor environments, it reduced these errors by 21.54% and 4.78%, respectively. These results demonstrate that the proposed controller provides superior robust tracking performance in unstructured outdoor environments.

## 1. Introduction

Driven by the rapidly aging population and structural shifts in industry, the demand for automation is escalating across diverse sectors, including logistics, construction, and agriculture [1]. While fixed industrial robots perform repetitive tasks in limited workspaces, mobile robots are attracting attention as a key means of next-generation automation due to their ability to respond flexibly across large work areas. In particular, wheeled mobile robots are widely used in various industrial sites based on their advantages such as superior energy efficiency, structural simplicity, and component compatibility.

However, existing steering mechanisms do not fully satisfy the requirements of diverse sites. Although Ackermann steering-based platforms excel in high-speed driving stability and energy efficiency, they are significantly constrained when navigating narrow passages or between dense facilities due to limitations in their turning radius. Skid-steer structures enable zero-radius turning but rely on slip between the wheels and the ground, which causes damage to tires and the terrain and leads to significant path tracking errors in low-friction or uneven environments [2]. Omni-wheels and Mecanum wheels allow for omnidirectional movement, but they have low efficiency due to their roller structure and are vulnerable to debris such as sand and mud, making them unsuitable for operation in outdoor environments [3].

To address these challenges, a 4-wheel independent steering (4WIS) mobile robot, which independently controls the steering of each wheel, has been proposed as an alternative. The 4WIS platform can implement a minimized turning radius and omnidirectional movement through front and rear wheel steering, and it can provide high traction in outdoor environments by using rubber tires. Furthermore, it has the advantage of enabling precise pose correction through the combination of driving force and steering angle for each wheel.

However, designing a model-based controller for a 4WIS mobile robot is difficult due to the nonlinearity caused by complex kinematics and wheel slip. Among existing studies, there are cases where the driving and steering torque of a 4WIS mobile robot are distributed using P controllers [4] or controlled via adaptive Fuzzy PID control [5]. However, PID control has the disadvantage of generating large path tracking errors in the presence of irregular disturbances and sharp curvature. Pure Pursuit-based methods have also been extended for 4WIS systems. Li et al. proposed an improved Pure Pursuit framework designed for four-wheel independent steering robots [6], and Yang et al. presented a constraint-oriented coordinated steering strategy for 4WIS vehicles [7]. Nevertheless, Pure Pursuit is fundamentally a geometric algorithm that lacks explicit slip compensation. Therefore, achieving robustness on slippery outdoor terrain requires additional compensation modules, which inevitably increases parameter sensitivity and tuning complexity. In the field of Model Predictive Control (MPC), studies have integrated the steering and velocity of 4WIS vehicles [8] or used Fuzzy logic to select appropriate driving modes and control speed via MPC [9]. However, MPC has limitations such as significant performance variations depending on the model and high computational costs. Among cases using nonlinear control, one study generated predictive output for the target path using feedforward control and overcame disturbances and uncertainty with Back-stepping Sliding Mode Control (SMC) [10]. However, this research was validated only in simulations and had the limitation of slow convergence speed for tracking errors. As such, the performance of model-based control can vary significantly depending on the kinematic model or human-designed rules, and tracking errors occur due to model uncertainty in environments with high nonlinearity. Additionally, complex algorithms make real-time control difficult due to high computational costs.

To overcome these limitations of model-based control, reinforcement learning (RL), which self-learns optimal policies through interaction with the environment, is gaining attention. RL has the advantage of solving control problems with strong nonlinearity without complex mathematical modeling of the system, leading to active research on driving control of mobile robots using RL. One study trained a basic RL policy for a skid-steer mobile robot in a simulator and performed supervised learning with driving data collected through actual robot operation [11]. However, it is unclear whether this approach is effective for mobile robots with steering structures. Another study used the Pure Pursuit algorithm for steering control and RL for speed control to verify a significant reduction in lateral error [12]. However, in actual robot experiments, performance degradation occurred compared to simulations due to communication and motor command delays. As such, for learning-based controllers, applying a policy learned in an ideal simulation environment directly to the real environment often leads to performance degradation due to the Sim-to-Real gap. Furthermore, research on RL-based path tracking for 4WIS mobile robots remains limited, and there are no cases systematically verifying performance in actual outdoor rough terrain environments.

To address this, this study proposes an RL-based controller for a 4WIS mobile robot that achieves robust path tracking performance even in outdoor rough terrain environments. The policy was trained in a dynamic simulator to ensure sample-efficient and safe learning before real-world deployment. The main contributions of this study are as follows:

1. We propose an RL-based path tracking algorithm for a 4WIS mobile robot capable of robustly responding to nonlinear dynamic characteristics.
2. We propose a method to minimize the Sim-to-Real gap by performing sensor and actuator domain randomization based on GPS and motor data from the real environment.
3. We validate the proposed method through simulation and real-world experiments on the developed 4WIS mobile robot, demonstrating superior path tracking performance compared to the Pure Pursuit algorithm in outdoor rough terrain environments.

The remainder of this paper is organized as follows: Section 2 describes the design of the 4WIS mobile robot, reinforcement learning, and empirical data-based domain randomization. Section 3 explains the existing path tracking controller and the experiments in simulation and real environments. Section 4 analyzes the experimental results. Finally, Section 5 presents the conclusion of the study.

## 2. Methodology

### 2.1. Design of 4-Wheel Independent Steering Mobile Robot

The 4-wheel independent steering (4WIS) mobile robot used in this study is designed with a structure that independently controls four driving motors and four steering motors. The main specifications of the robot are listed in Table 1. The platform dimensions are comparable to those of outdoor mobile robots widely used in prior robotics studies, such as the Clearpath Jackal UGV (Kitchener, ON, Canada) [13] and AgileX Scout Mini (Dongguan, China) [14]. Moreover, the selected wheel and motor specifications provide sufficient driving torque for operation on slopes of up to 10°. To ensure stable traction and shock absorption in outdoor environments, an independent suspension system was applied to each wheel module. The stiffness of the suspension was designed by considering the expected obstacle heights in the driving environment and the robot’s dynamic characteristics.

**Figure 1:**
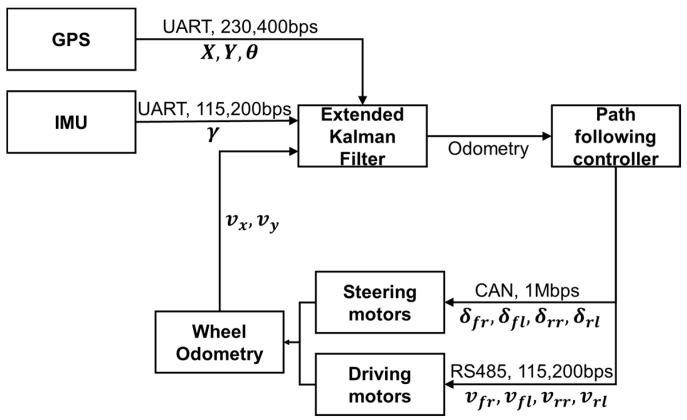
Hardware control architecture of the 4WIS mobile robot system.

**Figure 2:**
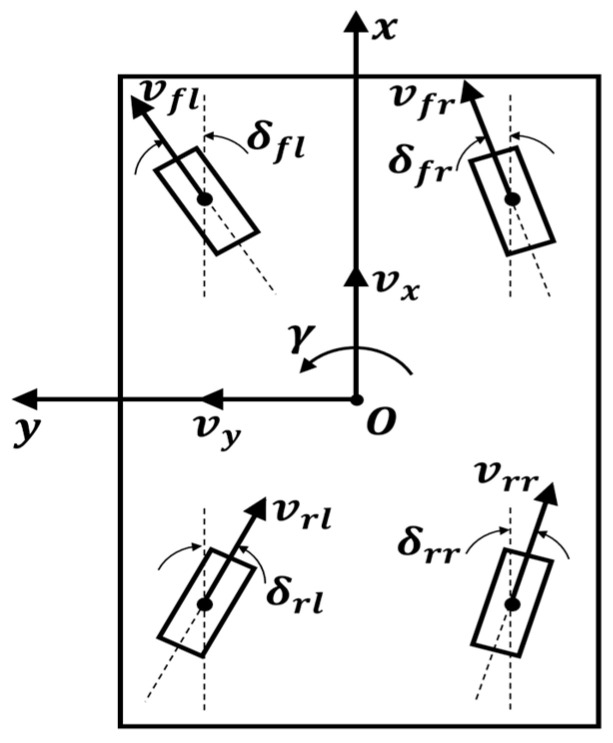
Kinematic model and variable definitions of the 4WIS mobile robot.

**Figure 3:**
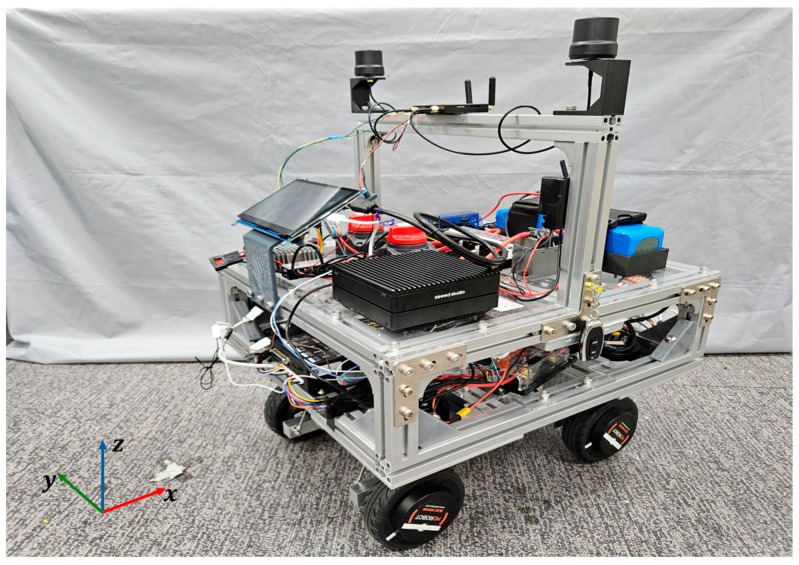
The 4-wheel independent steering mobile robot.

Figure 1 illustrates the communication and control diagram of the designed 4WIS mobile robot, while Figure 2 presents the robot’s geometric parameters. X,Y and θ denote the robot’s pose in the global coordinate system. $v_{x},v_{y}$ and γ represent the robot’s linear velocities and yaw rate, respectively. $\delta_{fr},\delta_{fl},\delta_{rr},\delta_{rl}$ correspond to the steering angles of each wheel, and $v_{fr},v_{fl},v_{rr},v_{rl}$ represent the speeds of each wheel. To achieve precise state estimation in outdoor environments, we utilized an RTK-based GPS receiver, an IMU, and wheel encoders on each wheel. The collected sensor data are fused using an Extended Kalman Filter (EKF) to estimate the robot’s real-time global position and pose. Figure 3 shows the developed 4WIS mobile robot.

### 2.2. Design of Reinforcement Learning-Based Path Tracking Controller

**Figure 4:**
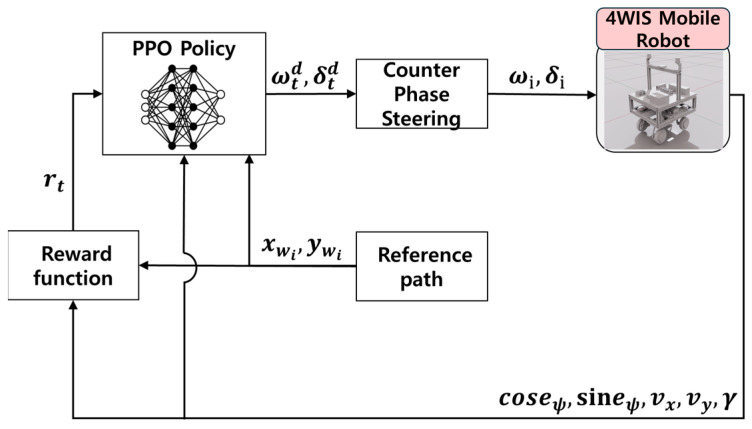
Schematic block diagram of the proposed RL-based path tracking control framework.

Figure 4 presents the overall framework of the proposed RL-based path tracking controller for the 4WIS mobile robot. $e_{\psi }$ denotes the heading error, and $x_{w_{i}}$ and $y_{w_{i}}$ represent the relative positions of future waypoints. Target path information obtained from the simulation environment and the robot’s state are used as inputs for the reward function and the policy network. The high-level RL policy then generates virtual commands, namely the virtual target drive angular velocity $\omega_{t}^{d}$ and the virtual target steering angle $\delta_{t}^{d}$. These are passed to a low-level counter phase kinematic controller [15], which distributes them into wheel-wise commands $\omega_{i}$ and $\delta_{i}$ for i∈{fr,fl,rr,rl}. The distributed commands are then applied to the drive and steering motors of the 4WIS robot.

**Figure 5:**
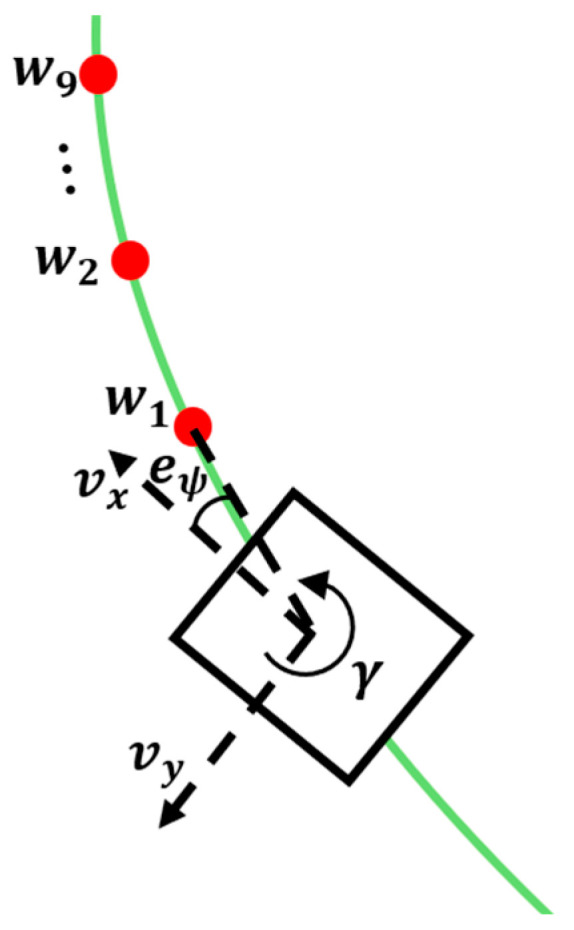
State space of Reinforcement learning in simulation.

Figure 5 shows the state space in the training environment. The state consists of a total of 25 dimensions, including the robot’s path information and kinematic state. The path information is defined as relative coordinates to future waypoints on the look-ahead path based on the robot’s local coordinate system. In this study, we selected nine specific look-ahead points located at 0.5, 1.0, 2.0, 3.5, 5.0, 7.5, 10.0, 12.5, and 15.0 m ahead of the robot. Waypoints were densely placed in the near-field region (0.5–3.5 m) to increase the immediate responsiveness of the control, while intervals were set wider in the far-field region to allow the policy to perceive the overall curvature shape of the path. Cosine and sine were applied to the heading error, which is the error between the path tangent direction and the robot’s heading, and included in the state. Through this, the discontinuity occurring every 2π cycle was removed. To reflect the dynamic characteristics of the robot, the robot’s current linear velocity and yaw rate were added to the state space, enabling indirect observation of the robot’s dynamic state according to environmental changes.

(1) $$r=w_{prog}r_{prog}+w_{align}r_{align}+w_{lat}r_{lat}+w_{head}r_{head}+w_{smooth}r_{smooth}$$

Equation (1) presents the reward function of the proposed controller. $r_{prog},r_{align},r_{lat},r_{head}$ and $r_{smooth}$ denote the progress reward, alignment reward, lateral error penalty, heading error penalty, and smoothness penalty, respectively, while $w_{prog},w_{align},w_{lat},w_{head}$ and $w_{smooth}$ represent the weights of each term. Table 2 shows the mathematical expression and weights for each reward function component. Here, $s_{t},v_{t},$ $u_{t}$, and ∆t represent the travel distance along the path, forward tangential speed along the path, steering command of the robot, and time step, respectively. $e_{y}$ and $e_{\psi }$ are the lateral error and heading error, while $\sigma_{y}$ and $\sigma_{\psi }$ are the scaling parameters, respectively. The reward function induces the agent to track the path quickly and accurately while driving smoothly. The progress reward is defined as the difference in travel distance along the path between the current step and the previous step; the agent receives a positive reward when it advances further compared to the previous step. The alignment reward is the product of the robot’s forward tangential speed along the path, the time step, a heading alignment gate, and a lateral error term. The factor $\max\;(0,cos(e_{\psi }))$ acts as a heading alignment, while $exp\left(-{\left(\frac{\left|e_{y}\right|}{\sigma_{y}}\right)}^{2}\right)$ smoothly attenuates reward as lateral error increases. Multiplication by Δt makes the term consistent across control frequencies. Therefore, $r_{\mathrm{align}}$ rewards fast forward progress only when the robot is both directionally aligned with the path tangent and close to the path centerline. The lateral error penalty and heading error penalty impose penalties as the lateral error and heading error increase, respectively. A quadratic function form was applied so that the penalty increases rapidly as the error grows. For the smoothness penalty, a penalty is imposed on the second-order difference in the steering command to prevent sudden changes in control input. This suppresses high-frequency oscillation of the steering angle and ensures driving stability.

The weights of the reward terms were determined heuristically through staged configuration. We first established $r_{prog}$ and $r_{align}$ to ensure forward progress and basic path-following behavior. Next, $r_{lat}$ was introduced to reduce steady-state cross-track error. Then, $r_{head}$ was added to suppress oscillatory behavior around the reference path. The scaling parameters $\sigma_{y}$ and $\sigma_{\psi }$ were introduced to normalize lateral and heading errors so that their penalties have comparable influence in the reward. Through iterative tuning, $\sigma_{y}=0.3$ and $\sigma_{\psi }=0.35$ were selected as a practical trade-off between tracking accuracy and convergence stability. Finally, $r_{smooth}$ was incorporated to reduce high-frequency steering oscillation. The final coefficients were selected to balance tracking accuracy and control smoothness in both simulation and outdoor tests.

### 2.3. Domain Randomization Based on Empirical Data

To ensure the learned policy robustly handles diverse path profiles, unstructured terrains and uncertainties in sensors and actuators, we applied three domain randomization techniques.

To secure path diversity, closed-loop paths with a radius of approximately 15 m were randomly generated in each episode using Catmull–Rom splines [16]. By initializing the agent at random positions along the generated path, it experiences diverse curvatures and geometric shapes during the training process.

To simulate ground irregularities, the terrain height in the simulation was randomly varied within a range of 0 to 10 cm. To reflect varying surface properties and traction conditions, the ground friction coefficient was domain randomized within the range of [0.3, 1.0], representing conditions from wet grass to dry asphalt. This enables the agent to acquire stable driving capabilities even in rough terrain environments characterized by frequent wheel slip and uneven surfaces.

**Figure 6:**
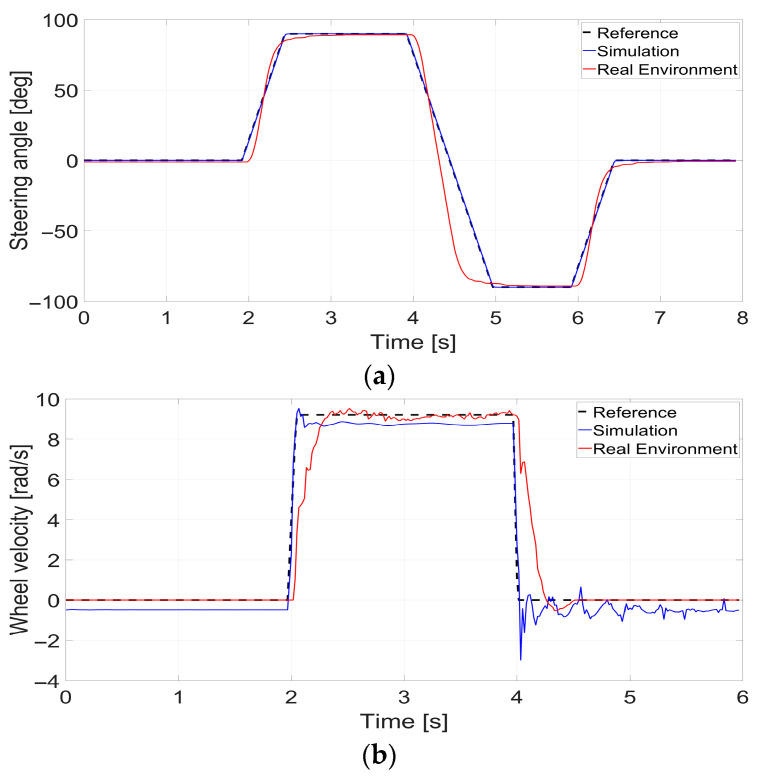
Comparison of motor response between simulation and physical hardware. (**a**) steering (**b**) driving.

To minimize the Sim-to-Real gap, we performed domain randomization based on empirical data from actual motors and GPS receivers. Unlike ideal simulation environments, actual motors exhibit response delays. Therefore, comparative experiments were conducted to quantify these delays. For the steering motors, position control was performed with the following sequence: 0° for the first 2 s, 90° from 2 to 4 s, −90° from 4 to 6 s, and 0° from 6 to 8 s. For the driving motors, velocity control was applied as follows: 0 rad/s from 0 to 2 s, 9.23 rad/s from 2 to 4 s, and 0 rad/s from 4 to 6 s. Here, 9.23 rad/s corresponds to the angular velocity of the driving motor when the robot moves forward at a speed of 0.6 m/s. Figure 6 presents a comparison of the response characteristics between the simulation model and the physical motor for identical control commands. The experimental results confirmed an average actuation delay of 40 ms in the physical system. Accordingly, during the reinforcement learning process, domain randomization was applied such that the steering motor error was randomly set within the range of −0.5° to 0.5°, and the motor delay within 10 to 40 ms for each episode.

**Figure 7:**
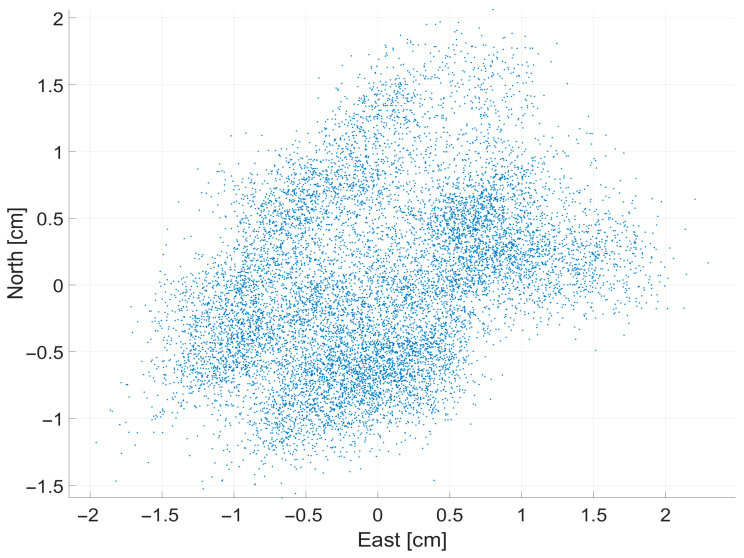
Dispersion of position data acquired via the GPS receiver.

**Figure 8:**
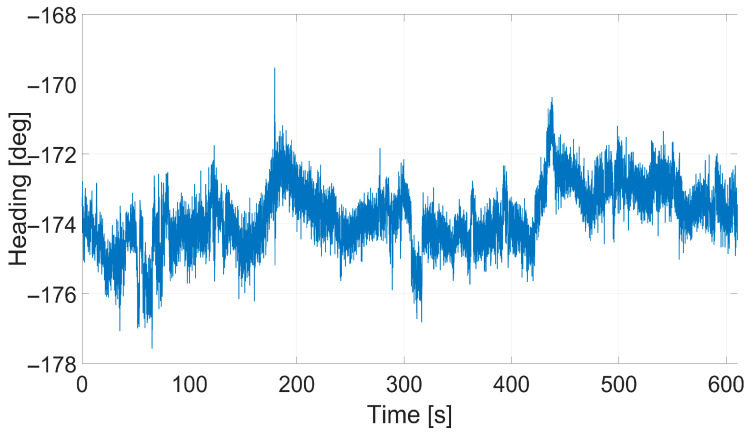
Time-series analysis of heading data collected by the GPS receiver.

Next, to measure the accuracy of the GPS receiver, position and heading data were collected for 10 min in a stationary state in an outdoor environment. Figure 7 shows the scatter plot of the position data acquired by the GPS receiver. Figure 8 displays the time-series GPS heading measurements. Table 3 summarizes the analytical results of the collected data. Finally, based on the analyzed empirical data, the domain randomization parameters for GPS measurements were set as shown in Table 4 to enhance the realism of the observation model.

## 3. Experiment

### 3.1. Experimental Scenarios

The reinforcement learning-based path tracking controller was trained using the Proximal Policy Optimization (PPO) algorithm [17] within Isaac Sim(v4.5.0), utilizing the Isaac Lab framework(v2.0.1) [18]. The hyperparameters used for reinforcement learning are listed in Table 5. The policy/value network was set to [256, 256] to provide sufficient model capacity for the 25-dimensional state space and nonlinear outdoor dynamics. The discount factor and learning rate were retained as stable default values in Isaac Lab PPO training examples. The number of parallel environments was selected considering the computational and memory limits of the RTX 3060 platform. Training was stopped at 59 M timesteps when the loss trends and policy performance reached a stable plateau.

To verify the path tracking performance, experimental scenarios were established in both actual outdoor environments and a simulation environment mimicking rough terrain. For comparison, the geometric Pure Pursuit controller [19] was selected as the baseline. This choice was made because the Pure Pursuit algorithm is widely used for path tracking in mobile robots operating at low speeds and on paths with high curvature. The reference path was generated using GPS data collected by manually driving the robot in the outdoor environment. In all experiments, the robot’s driving speed was set to 0.6 m/s.

In the simulation environment, the ground friction coefficient was set to 0.35, and rough terrain was simulated by generating terrain with height variations ranging from 0 to 3 cm. For the real-world experiments, tests were conducted on two types of terrain.

**Figure 9:**
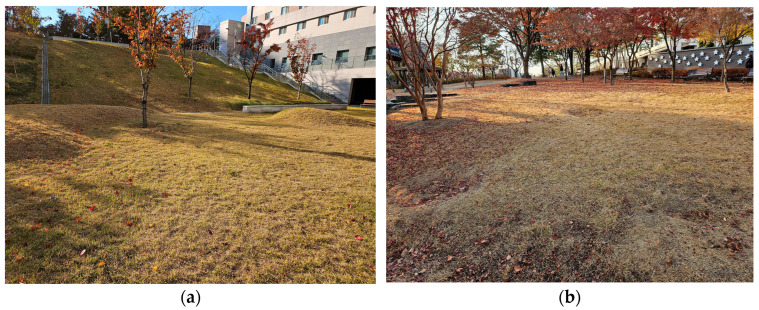
Field test environments for outdoor performance evaluation. (**a**) Grass, (**b**) Sloped grass.

Figure 9 presents photographs of each outdoor environment. The grass terrain consists of grass with an average length of 4 cm and exhibits minimal elevation changes. The sloped grass terrain features an average slope of 10 degrees and contains irregularities along the path, representing a terrain with high nonlinearity. The evaluation metrics were defined as the Root Mean Square Error (RMSE) and maximum values of the robot’s lateral error and heading error relative to the reference path.

### 3.2. Simulation Results

**Figure 10:**
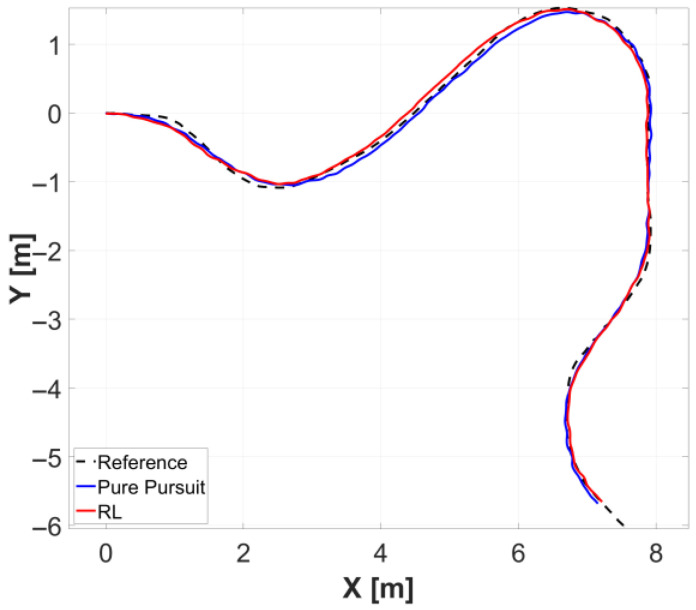
Comparative analysis of trajectory tracking performance for each controller relative to the reference path in simulation.

**Figure 11:**
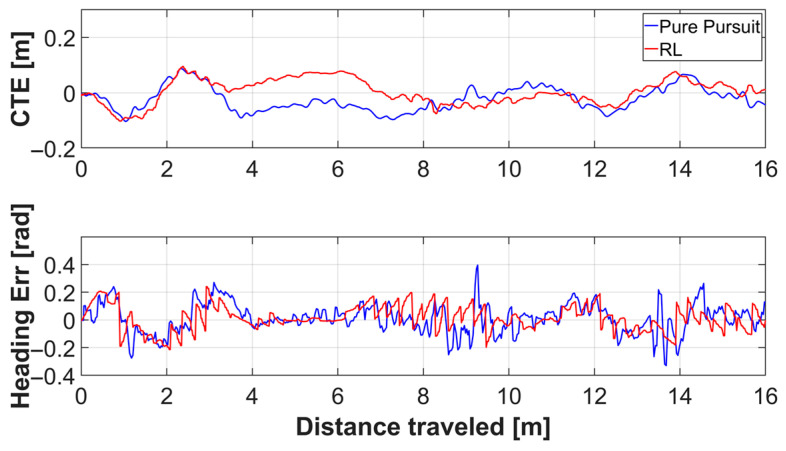
Plots of cross track error and heading error of each controller corresponding to the distance traveled in a simulation environment.

Figure 10 illustrates the trajectories of each controller on the x-y plane. Figure 11 presents the trends of lateral error and heading error for each controller with respect to the traveled distance. Table 6 summarizes the experimental results for each controller. The reinforcement learning (RL) controller demonstrated a slight reduction in lateral error and a significant reduction in heading error compared to the Pure Pursuit controller.

### 3.3. Outdoor Experiment Results

**Figure 12:**
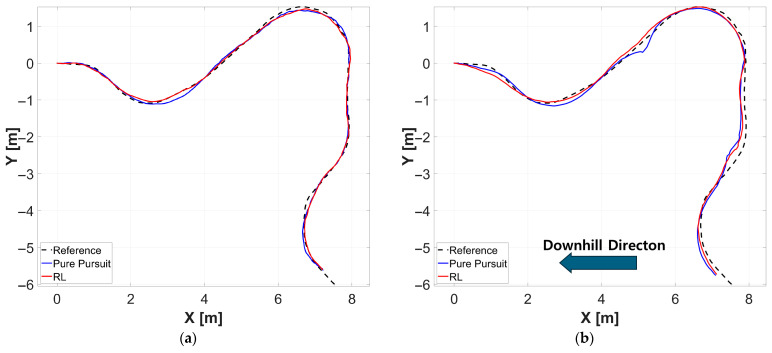
Comparative analysis of trajectory tracking performance for each controller relative to the reference path in a real environment. (**a**) Grass (**b**) Sloped grass.

**Figure 13:**
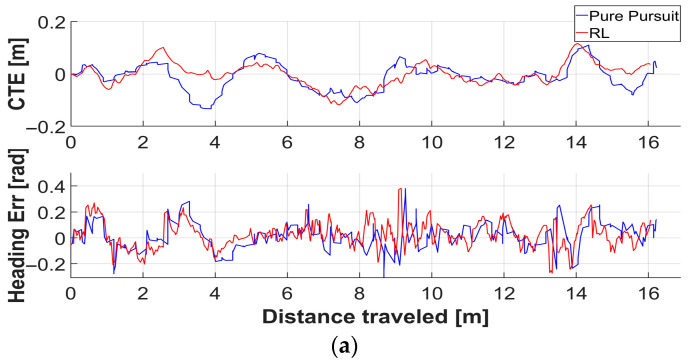

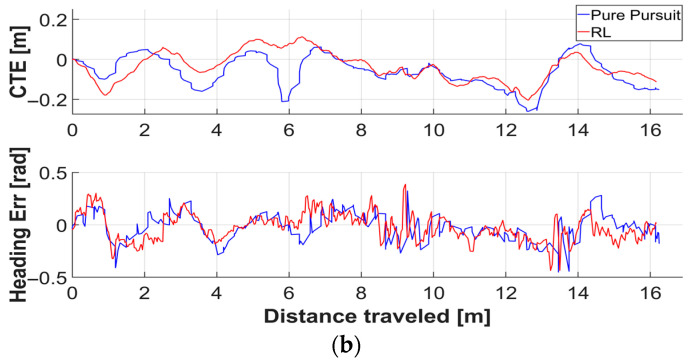
Plot of cross track error and heading error of each controller corresponding to the distance traveled in real environment. (**a**) Grass, (**b**) Sloped grass.

Figure 12 depicts the trajectories of each controller in the outdoor experiments. Figure 13 illustrates the lateral and heading error trends for each controller corresponding to the traveled distance in both grass and sloped grass environments. Table 7 presents the experimental results for each outdoor environment and controller. In both the grass and sloped grass environments, the RL controller exhibited lower errors across all evaluation metrics compared to the Pure Pursuit controller.

## 4. Discussion

In both simulation and real-world environments, the performance of the Pure Pursuit controller significantly degraded due to uneven terrain and wheel slip. In particular, on the sloped grass terrain, it was observed that both controllers drifted toward the downslope direction relative to the reference path due to slip. However, the proposed reinforcement learning controller exhibited a smaller lateral error compared to the Pure Pursuit controller. This is attributed to the fact that the reinforcement learning policy indirectly infers environmental dynamic changes through the robot’s state information and effectively responds to nonlinear disturbances by correcting steering and driving commands in real-time. Consequently, the reinforcement learning controller effectively overcame the system’s nonlinearities and demonstrated superior performance over the Pure Pursuit controller across all evaluation metrics.

In future research, we plan to implement curriculum-based reinforcement learning with gradually increasing terrain complexity and refined reward functions to further minimize lateral and heading errors across diverse terrains.

## 5. Conclusions

This paper proposes a reinforcement learning-based path tracking controller to overcome the structural limitations of existing omnidirectional mobile robots and achieve robust path tracking performance, even in complex outdoor environments. To this end, we designed a 4-wheel independent steering (4WIS) mobile robot designed for outdoor rough terrain traversal. We implemented a dynamic model of the 4WIS robot in a simulator and trained a reinforcement learning agent based on the PPO algorithm. To ensure policy robustness, we applied path and terrain randomization, as well as domain randomization techniques to bridge the Sim-to-Real gap. To validate the performance of the proposed controller, comparative experiments were conducted against the Pure Pursuit method, a conventional path tracking controller, in both dynamic simulation and real-world environments. Experimental results demonstrated that the proposed controller achieved a 21.54% improvement in lateral error RMSE and a 4.78% improvement in heading error RMSE compared to the Pure Pursuit controller in highly nonlinear environments, such as sloped grass terrain, thereby proving its robust path tracking capability.

## Figures and Tables

**Table 1:** Specifications and Physical Parameters of the Developed 4WIS Mobile Robot.

Specification	Value/Details	
Dimension	50 cm × 46 cm × 70 cm	
Weight	30 kg	
Max speed	1.0 m/s	
DOF	Motion	3 DOF
	Actuated	8 DOF
Sensors	GPS, IMU, Motor encoders	

**Table 2:** Mathematical Expression and Weights of a Reward Function.

	Mathematical Expression	Weights
$r_{prog}$	$max\;(0,\;s_{t}-s_{t-1})$	20.0
$r_{align}$	$v_{t}\cdot ∆t\cdot max(0,\;cos\;(e_{\psi }))\cdot exp\left(-{\left(\frac{\left|e_{y}\right|}{\sigma_{y}}\right)}^{2}\right)$	16.0
$r_{lat}$	$-{\left(\frac{\left|e_{y}\right|}{\sigma_{y}}\right)}^{2}$	0.8
$r_{head}$	$-{\left(\frac{\left|e_{\psi }\right|}{\sigma_{\psi }}\right)}^{2}$	0.2
$r_{smooth}$	$-{\left({∆}^{2}u_{t}\right)}^{2}$	0.2

**Table 3:** Statistical Analysis of GPS Experimental Data.

Average Data Update Rate	20 Hz
Horizontal position noise (1σ)	0.007 m (N: 0.0065 m, E: 0.0073 m)
Horizontal error (95%)	0.016 m (max 0.023 m)
Heading noise (1σ)	0.9°

**Table 4:** Domain Randomization Parameters Based on GPS experiment.

GPS Data Update Rate	17–23 Hz
GPS latency	30–120 ms
Position noise	0.04–0.10 m
Position bias	0–0.01 m
Heading noise	0.3°–2.0°
Heading bias	−1°–1°
GPS dropout	0–5%

**Table 5:** Hyperparameters of Reinforcement Learning.

Policy/Value Network	[256, 256], Activation: Elu
Discount Factor	0.99
Learning Rate	5.0 × 10^−4^
Total Timesteps	59 M
Parallel Environments	4096
Training Duration	18 min

**Table 6:** Metrics Comparison of Each Controller in a Simulation.

Metric		Pure Pursuit	RL
CTE (m)	RMSE	0.0475	0.0445
	Max	0.1039	0.1021
Heading error (rad)	RMSE	0.1075	0.0903
	Max	0.3958	0.2404

**Table 7:** Metrics Comparison of Each Controller in a Real Environment.

Metric	Grass		Sloped Grass	
	Pure Pursuit	RL	Pure Pursuit	RL
CTE RMSE (m)	0.0509	0.0424	0.1026	0.0805
CTE Max (m)	0.1327	0.1178	0.2597	0.2041
Heading RMSE (rad)	0.1089	0.0996	0.1340	0.1276
Heading Max (rad)	0.3796	0.3795	0.4507	0.4352

## Data Availability

The data gathered during this study are available upon request from the first author.

## Abbreviations

The following abbreviations are used in this manuscript:

RL	Reinforcement Learning
4WIS	4-wheel independent steering
PP	Pure Pursuit
MPC	Model Predictive Control
